# Developmental, cellular and biochemical basis of transparency in clearwing butterflies

**DOI:** 10.1242/jeb.237917

**Published:** 2021-05-28

**Authors:** Aaron F. Pomerantz, Radwanul H. Siddique, Elizabeth I. Cash, Yuriko Kishi, Charline Pinna, Kasia Hammar, Doris Gomez, Marianne Elias, Nipam H. Patel

**Affiliations:** 1 Department of Integrative Biology, University of California, Berkeley, Berkeley, CA 94720, USA; 2 Marine Biological Laboratory, Woods Hole, MA 02543, USA; 3 Image Sensor Lab, Samsung Semiconductor, Inc., 2 N Lake Ave. Ste. 240, Pasadena, CA 91101, USA; 4 Department of Medical Engineering, California Institute of Technology, Pasadena, CA 91125, USA; 5 Department of Environmental Science, Policy, & Management, University of California Berkeley, Berkeley, CA 94720, USA; 6 Department of Molecular & Cell Biology, University of California, Berkeley, Berkeley, CA 94720, USA; 7 Department of Biology and Biological Engineering, California Institute of Technology, Pasadena, CA 91125, USA; 8 ISYEB, 45 rue Buffon, CP50, 75005, Paris, CNRS, MNHN, Sorbonne Université, EPHE, Université des Antilles, France; 9 CEFE, 1919 route de Mende, 34090, Montpellier, CNRS, Université Montpellier, Université Paul Valéry Montpellier 3, EPHE, IRD, France

**Keywords:** Anti-reflection, Nanostructures, Glasswing, Lepidoptera, Cytoskeleton, Morphogenesis

## Abstract

The wings of butterflies and moths (Lepidoptera) are typically covered with thousands of flat, overlapping scales that endow the wings with colorful patterns. Yet, numerous species of Lepidoptera have evolved highly transparent wings, which often possess scales of altered morphology and reduced size, and the presence of membrane surface nanostructures that dramatically reduce reflection. Optical properties and anti-reflective nanostructures have been characterized for several ‘clearwing’ Lepidoptera, but the developmental processes underlying wing transparency are unknown. Here, we applied confocal and electron microscopy to create a developmental time series in the glasswing butterfly, *Greta oto*, comparing transparent and non-transparent wing regions. We found that during early wing development, scale precursor cell density was reduced in transparent regions, and cytoskeletal organization during scale growth differed between thin, bristle-like scale morphologies within transparent regions and flat, round scale morphologies within opaque regions. We also show that nanostructures on the wing membrane surface are composed of two layers: a lower layer of regularly arranged nipple-like nanostructures, and an upper layer of irregularly arranged wax-based nanopillars composed predominantly of long-chain *n*-alkanes. By chemically removing wax-based nanopillars, along with optical spectroscopy and analytical simulations, we demonstrate their role in generating anti-reflective properties. These findings provide insight into morphogenesis and composition of naturally organized microstructures and nanostructures, and may provide bioinspiration for new anti-reflective materials.

## Abstract (precis)

**Summary:** Transparency is a fascinating, yet poorly studied, optical property in living organisms. We elucidated the developmental processes underlying scale and nanostructure formation in glasswing butterflies, and their roles in generating anti-reflective properties.

## INTRODUCTION

The wings of butterflies and moths (Lepidoptera) have inspired studies across a variety of scientific fields, including evolutionary biology, ecology and biophysics (Beldade and Brakefield, 2002; Prum et al., 2006; Gilbert and Singer, 1975). Lepidopteran wings are generally covered with rows of flat, partially overlapping scales that endow the wings with colorful patterns. Adult scales are chitin-covered projections that serve as the unit of color for the wing. Each scale can generate color through pigmentation via molecules that selectively absorb certain wavelengths of light, structural coloration, which results from light interacting with the physical nanoarchitecture of the scale; or a combination of both pigmentary and structural coloration (Stavenga et al., 2014; Thayer et al., 2020). Cytoskeletal dynamics, including highly organized F-actin filaments during scale cell development, play essential roles in wing scale elongation and prefigure aspects of scale ultrastructure (Dinwiddie et al., 2014; Day et al., 2019).

**Fig. 1.**
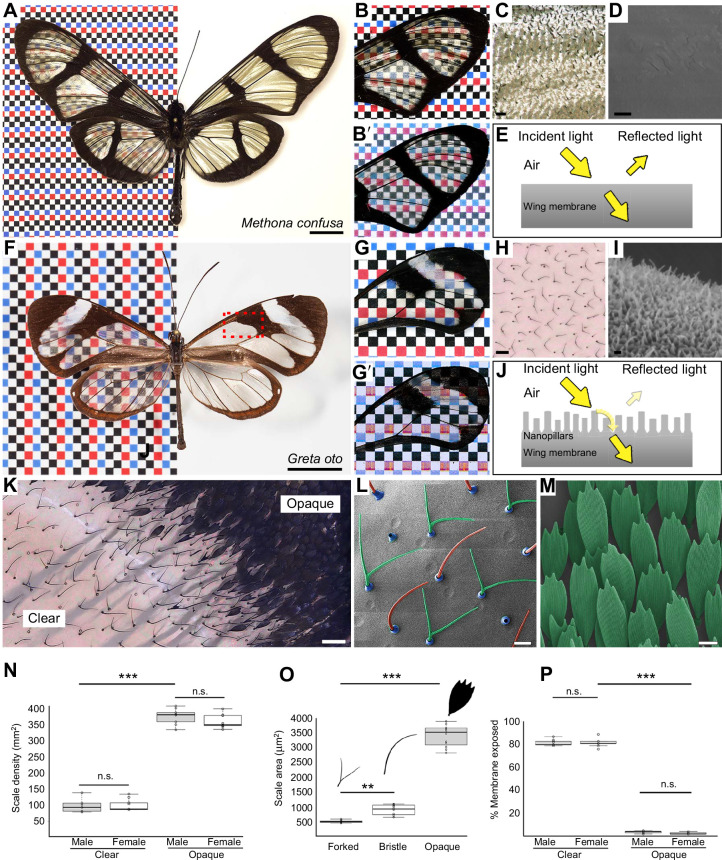
**Examples of clearwing butterflies, and wing scale features in *Greta oto.*** (A) Giant glasswing *Methona confusa* (Nymphalidae: Ithomiini). Scale bar, 1 cm. Wings under (B) reflected and (B′) transmitted light, illustrating general transparency, but strong light reflectance off the wing surface. (C) High magnification of the clear wing region, showing reflection off the membrane surface. Scale bar, 100 μm. (D) Scanning electron microscopy (SEM) of the wing membrane demonstrates that the surface is smooth and devoid of nanostructures. Scale bar, 1 μm. (E) Simplified diagram of reflection and transmission on the smooth wing membrane of *M. confusa*. Owing to the higher refractive index of the wing membrane, light is reflected at the surface. (F) Glasswing *G. oto* (Nymphalidae: Ithomiini). The red box indicates the representative clear and opaque dorsal forewing regions investigated in this study. Scale bar, 1 cm. Wings under (G) reflected and (G′) transmitted light and (H) high magnification of the clear wing region, illustrating minimal reflectance. Scale bar, 100 μm. (I) SEM of the wing membrane surface reveals irregularly sized nanopillars that enable omnidirectional anti-reflective properties (Siddique et al., 2015). Scale bar, 200 nm. (J) Simplified diagram of reflection and transmission on the wing of *G. oto* containing wing surface nanostructures, which reduce reflection by creating a smoother gradient of refractive indices between air and chitin. (K) High magnification of a transition boundary between a clear and opaque wing region. Scale bar, 100 μm. (L) SEM of adult scales in a clear wing region of *G. oto*, revealing alternating forked (green false coloring) and bristle-like (red false coloring) scale morphologies (socket false colored in blue). Scale bar, 20 μm. (M) SEM of scales in an opaque wing region, highlighting typical large, flat scale morphologies. Scale bar, 20 μm. (N) Measurements of scale density in clear and opaque wing regions, (O) scale surface area for forked, bristle-like, and opaque scale morphologies, and (P) percent of wing membrane exposed in *G. oto* clear and opaque regions. Error bars indicate means+s.d. of three measurements taken from wings in three different individuals (*P*-values based on *t*-tests for N and O, and ANOVA for P; ***P<0.001; **P<0.01).

In contrast to typical colorful wings, numerous species of butterflies and moths possess transparent wings that allow light to pass through, so that objects behind them can be distinctly seen (Fig. 1A–H) (Goodwyn et al., 2009; Yoshida et al., 1997; Siddique et al., 2015). This trait has been interpreted as an adaptation in the context of camouflage, in which some lineages evolved transparent wings as crypsis to reduce predation (Arias et al., 2019; 2020; Mcclure et al., 2019). Transparency results from the transmission of light across the visible spectrum through a material, in this case the chitin membrane, without appreciable absorption or reflection. Levels of reflection are largely determined by the differences in refractive indices between biological tissues and the medium, and a larger difference results in higher surface reflection. Previous studies on transparency in nature have primarily focused on aquatic organisms, which are frequently transparent, aided by the close match between the refractive indices of their aqueous tissue and the surrounding medium – water (e.g. Johnsen, 2001). By contrast, transparency is rare and more challenging to achieve on land, primarily owing to the large difference between the refractive indices of terrestrial organism's tissue (*n*=∼1.3–1.5) and air (*n*=1), which results in significant surface reflection (Yoshida et al., 1997; Johnsen, 2014; Bagge, 2019).

Nevertheless, some organisms have evolved morphological innovations that overcome the challenges of terrestrial transparency, notably in the form of anti-reflective nanostructures. Early experiments elucidated highly ordered sub-wavelength nanostructures (termed ‘nipple arrays’) on the corneal surface of insect eyes (Bernhard, 1962). These structures were found to generally be ∼150–250 nm in height and spaced ∼200 nm apart, which reduces reflection across a broad range of wavelengths by creating a smoother gradient of refractive indices between air and chitin (Stavenga et al., 2006). Nanostructure arrays have also been identified on the wings of cicadas, which help to reduce surface reflection over the visible spectrum (Huang et al., 2015).

Some lepidopterans possess ‘clear wings’ in which scales have undergone modifications that enable light to reach the wing membrane surface. The wing itself is composed of chitin and has some inherent transparency, but owing to the high refractive index of chitin, *n*=1.56, the wing surface reflects light (Vukusic et al., 1999). For example, the butterfly *Methona confusa* (Nymphalidae: Ithomiini) has exposed wing membrane that lacks nanostructures on the surface, and as a result, the wing is somewhat transparent, but retains a high degree of reflectivity (Fig. 1A–E). Conversely, the longtail glasswing, *Chorinea faunus* (Riodinidae), contains small, widely spaced scales and dome-shaped chitin nanoprotuberances on the membrane that generate anti-reflective properties (Narasimhan et al., 2018). The hawkmoth, *Cephonodes hylas* (Sphingidae), has nude wings owing to deciduous scales that fall out upon eclosion, and possesses anti-reflective nanostructures on its wing surface that morphologically resemble insect corneal nipple arrays (Yoshida et al., 1997). Nipple array nanostructures have also been characterized in transparent wing regions of the tiger moth *Cacostatia ossa* (Erebidae) (Deparis et al., 2014). Finally, the glasswing butterfly, *Greta oto* (Nymphalidae: Ithomiini), contains thin, vertically oriented scales, allowing the wing surface to be exposed, along with nanopillars that coat the surface (Fig. 1F–J). These irregularly arranged nanopillars feature a random height distribution and enable omnidirectional anti-reflective properties (Fig. 1I,J) (Siddique et al., 2015; Binetti et al., 2009). More recent studies have explored aspects of structural diversity, optical properties, phylogenetic distribution and ecological relevance of transparency within a wide range of butterflies and moths, highlighting that transparency has evolved multiple times independently and may present evolutionary benefits (Mcclure et al., 2019; Gomez et al., 2020 preprint; Pinna et al., 2020 preprint).

Lepidoptera are proving to be an excellent group to investigate transparency on land, but the developmental processes underlying wing transparency are currently unknown. This presents a gap in our understanding of lepidopteran wing evolution and diversification, as transparent butterflies and moths contain multitudes of intriguing scale modifications and sub-wavelength cuticular nanostructures (Gomez et al., 2020 preprint; Pinna et al., 2020 preprint). Therefore, we set out to explore the development of wing transparency in the glasswing butterfly, *G.* *oto*, which belongs to a diverse tribe (∼393 species) of predominantly transparent neotropical butterflies (Elias et al., 2008). We applied confocal and transmission electron microscopy (TEM) to compare wing development, scale cytoskeletal organization and membrane surface nanostructures between clear and opaque wing regions. Using chemical treatments, scanning electron microscopy and gas chromatography–mass spectrometry, we found that nanostructures on the wing membrane surface are composed of two layers: a lower layer of chitin-based nipple-like nanostructures, and an upper layer of wax-based nanopillars composed predominantly of long-chain *n*-alkanes. Finally, by removing wax-based nanopillars, we demonstrate their role in dramatically reducing reflection on the wing surface via optical spectroscopy and analytical simulations.

## MATERIALS AND METHODS

### Samples

Glasswing butterfly [*Greta oto* (Hewitson 1854)] pre-pupae were purchased from Magic Wings Butterfly House (Deerfield, MA, USA) and reared on *Cestrum nocturnum* (Solanaceae) leaves at 27°C and 60% humidity on a 16 h:8 h light:dark cycle at the Marine Biological Laboratory (Woods Hole, MA, USA) under United States Department of Agriculture permit number P526P-19-02269. At the appropriate time of development, pupal wings were dissected and age was recorded as hours after pupal case formation (h APF) Dinwiddie et al. (2014). The average timeline from pupation to eclosion (adult emergence) for *G. oto* at 27°C is approximately 7 days, and we report our time series here which covers early aspects of wing scale development.

### Optical imaging and scale measurements

Images of whole-mounted specimens were taken with a Canon EOS 70D digital camera with an EF 100 mm f/2.8 L macro lens. High-magnification images of disarticulated wings were taken with a Keyence VHX-5000 digital microscope. Scale density was determined by counting the numbers of scales in a 1 mm^2^ area. Scales were also removed from the wings and laid flat onto a slide, and Keyence software was used to measure the surface area of individual scales. Images of clear and opaque regions were processed with Keyence software to measure the percentage of area covered by scales. We took measurements from three individual males and three individual females that were reared in the same cohort. All measurements were taken on the dorsal surface of the forewing (indicated by the red box in Fig. 1F) and each measurement was replicated three times per individual. For statistics, we used *N*=3, where measurements for each individual were averaged and the difference between each wing measurement group (scale density in clear versus opaque regions and percent wing membrane exposed in clear versus opaque regions) was analyzed using *t*-tests for two independent samples with unequal variance estimates. An ANOVA test was used to analyze scale area measurements between different scale morphologies (bristle, forked and opaque).

### Confocal microscopy

For confocal microscopy of fixed tissue, pupal wings were dissected and fixed in PEM buffer (0.1 mol l^−1^ PIPES, 2 mmol l^−1^ EGTA, 1 mmol l^−1^ MgSO_4_, pH 6.95) with 3.7% paraformaldehyde for 20–30 min at room temperature, as described previously (Dinwiddie et al., 2014). Fixed wings were incubated in 1X PBS+0.1% Triton-X 100 (PT) with 1:200 dilution of phalloidin, Alexa 555 conjugated (Invitrogen A34055), and wheat germ agglutinin, Alexa 647 conjugated (Invitrogen W32466) at a dilution of 1:200 overnight at 4°C. Wings were washed in PT and then placed in 50% glycerol:PBS with 1 µg ml^−1^ DAPI overnight at 4°C. Wing samples were placed on microscope slides and mounted in 70% glycerol:PBS. A coverslip (#1.5 thickness) was applied, and each preparation was sealed around the edges with nail polish. Slides of fixed tissue were examined with an LSM 880 confocal microscope (Carl Zeiss, Germany) with 40× and 63× objectives. Confocal images and movies were generated using Imaris Image Analysis Software (Bitplane, Oxford Instruments, UK).

### Scanning electron microscopy

We cut 2 mm square pieces from dry wings, coated them with a 10 nm layer of gold using the Bio-Rad E5400 Sputter Coater, and imaged with a Hitachi TM-1000 SEM at 5 kV. Top-view and cross-section SEM images were analysed with ImageJ 1.52 to measure membrane thickness and nanostructure dimensions (*n*=6 individuals).

### Transmission electron microscopy

For TEM, wings of *G.* *oto* pupae were dissected and fixed in 2% glutaraldehyde, 2% paraformaldehyde in 0.1 mol l^−1^ sodium cacodylate buffer overnight at 4°C (pH 7.4). Samples were then rinsed in 0.1 mol l^−1^ cacodylate buffer (pH 7.4) and post-fixed in 1% aqueous osmium tetroxide in 0.1 mol l^−1^ cacodylic buffer overnight at 4°C, then rinsed in water. Samples were en bloc stained with 1% uranyl acetate in water and then rinsed in water. Samples were dehydrated through a graded ethanol series (50–100% in 10% steps), rinsed in propylene oxide, and then infiltrated in 50% resin and propylene oxide overnight. Samples were infiltrated with Epon/Alardite embedding medium (70%, 80%, 95% to 100% steps) and polymerized at 60°C for 2 days. Thin sections (∼70 nm) were cut on an Ultramicrotome RMC PowerTome XL using a Diatome diamond knife. Digital images were taken using a JEOL 200 transmission electron microscope (JEOL, USA).

### Wing surface wax extraction and analysis

To identify the molecular composition of the transparent wing surface, we pooled forewing dissections from three individual adults and performed two replicates for chloroform-based extractions and two replicates for hexane-based extractions (after Futahashi et al., 2019). First, the samples were soaked with 100 µl of either hexane or chloroform and gently mixed for 15 min on a Thermolyne RotoMix 51300. The liquid solutions containing dissolved wing surface compounds were then transferred to glass vials with fixed microvolume inserts, and the solvent was evaporated under a stream of high-purity nitrogen gas (99.99%). Dried extracts were re-dissolved in fixed volumes of hexane (10 µl), and half of the extract (5 μl) was injected by automatic liquid sampler into a gas chromatograph coupled with a mass selective detector (GC: 7890A; MS: 5975C; Agilent Technologies, USA) operating in electron impact mode. The injection was performed in a split/splitless injector in the splitless mode. Separation of compounds was performed on a fused silica capillary column (DB-5MS, 30 m×0.32 mm×0.25 μm, Agilent J&W GC columns, USA) with a temperature program starting from 80°C for 5 min and increasing by 80°C min^−1^ to 200°C, followed by an increase of 5°C min^−1^ to 325°C, which was held for 3 min, with helium used as the carrier gas, positive electron ionization (70 eV), analog to digital (A/D) sampling rate was set at 4, and the scan range was m/z 40.0 to 650.0. Chemical data processing was carried out using the software Enhanced Chemstation (Agilent Technologies). We retained peaks with abundances greater than 0.25% of the total and compounds were identified according to their retention indices, diagnostic ions and mass spectra, which are provided in Table S1. For some peaks, it was not possible to narrow the identity to a single specific compound because (1) some low abundance substances produced poor quality mass spectra, (2) multiple compounds could have produced the observed fragmentation patterns and/or (3) multiple compounds may have co-eluted at the same retention time.

### Optical measurements

The wing reflection measurements were performed on a Cary 5000 UV-Vis-NIR spectrophotometer, equipped with a light source of tungsten halogen and an integrating sphere diffuse reflectance accessory (Internal DRA 1800). Wing measurements from the dorsal wing surface were recorded using three different individuals for control treatments (untreated) and three different individuals for hexane treatments with unpolarized light with a spot size of 100 µm for an incident angle of 8 deg to avoid the loss of direct specular reflectance component through the aperture. All measurements were taken in the dark to avoid possible stray illumination from the surrounding environment and we performed two technical replicates for each individual wing. A reference measurement was done with a calibrated commercial white spectralon standard to calculate the relative diffuse reflectance. The reflectance measurements and mean data are available from Dryad (https://doi.org/10.6078/D1TD7H).

### Optical simulations

The total volume fraction of the untreated wing along the height *h* can be given by:

**Table:**
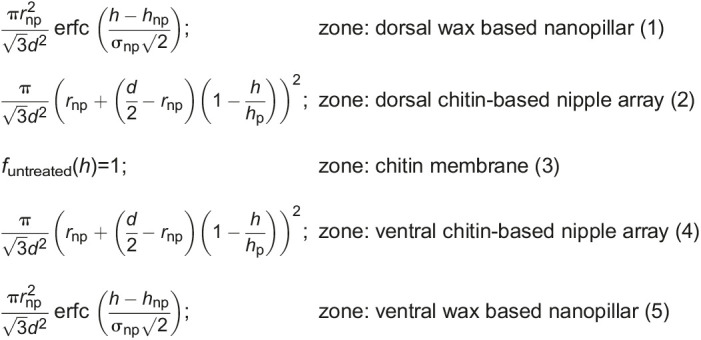

where 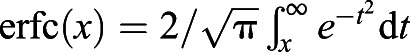 is the complementary error function. The average distance between two nanostructures is represented as *d*, conical shaped cuticular nipple nanostructure height as *h*_p_, wax-based irregular nanopillar radius as *r*_np_, mean height of the irregular nanopillar distribution as *h*_np_ and their corresponding variance as σ_np_.

The volume fraction of the treated wing without the irregular nanopillars will be:

**Table:**
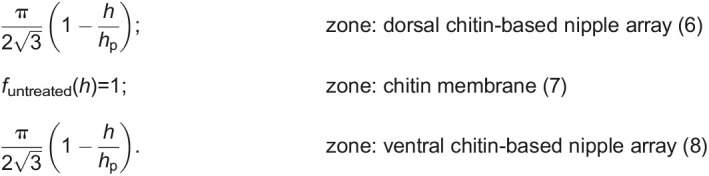

After determining the volume fraction, the corresponding refractive index changes along the wing at any height *h* were calculated using the effective medium theory (EMT) with the Maxwell–Garnett approximation as shown in Fig. 6E (see Fig. S2). EMT pertains to analytical or theoretical modeling that describes the macroscopic properties of subwavelength nanostructured materials, when the nanostructures collectively affect the optical properties. EMT is developed from averaging the multiple values of the constituents that directly make up the nanostructured material including the surrounding media, in this case, chitin, wax and air. The refractive indices of the different materials were considered as *n*_air_=1, *n*_chitin_=1.56+*i*0.008 (Vukusic et al., 1999; Narasimhan et al., 2018), and we considered *n*_wax_=1.39 (based on Hooper et al., 2006). Therefore, the effective refractive index *n*_eff_ can be calculated for any *h* using the equations below with the calculated volume fractions, where air volume fraction can be calculated by corresponding *f*_air_=1−*f*_wax/chitin_:

**Table:**
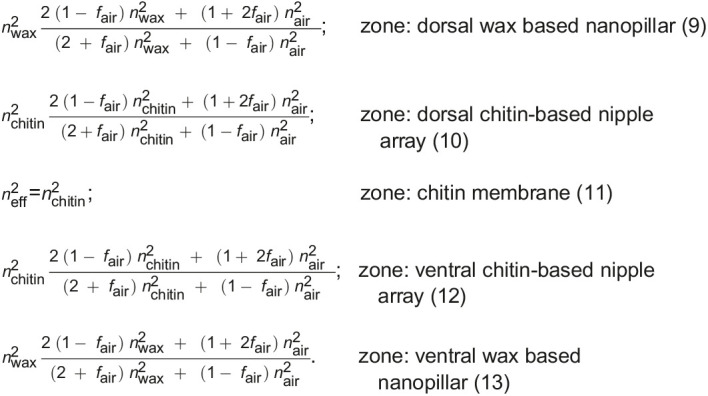

Afterwards, the transfer matrix method (TMM) computed the reflectance from the stratified medium with calculated refractive index profiles as shown in Fig. 6E for the unpolarized condition (taking the average of both s- and p-polarization) at an incident angle of 8 deg (to replicate the experimental condition). The basic formalism of TMM relies on the calculation of thin film reflection and transmission from Maxwell's electromagnetic equations using the boundary conditions. Because of the stack of thin films, the reflectance and transmittance is calculated with a transfer matrix formalism describing the propagation of light from layer to layer. The membrane-only reflection at normal incident light can be directly calculated from Siddique et al. (2016):(14)
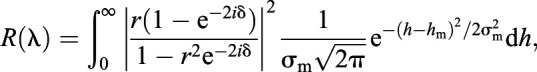
where membrane thickness is *h*_m_ and modulation is σ_m_, δ=(2π*n*_chitin_*h*)*/*λ is the phase delay introduced by the membrane thickness of *h*, and *r* is the reflection coefficient at the air–chitin boundary governed by Fresnel's equation for a normal incident light, i.e. *r=*(1−*n*_chitin_)/(1+*n*_chitin_).

## RESULTS

### Scale measurements in clear and opaque wing regions of adult *G. oto*

We investigated features of scale density, scale morphology and the amount of wing surface exposed in adult *G.* *oto*. We focused on two adjacent regions on the dorsal surface of the forewing for consistency: a clear region within the discal cell and an opaque region that consists mainly of black scales near the cross-vein (indicated by the red box in Fig. 1F). The clear wing region contained two types of alternating scale morphologies – bristle-like scales and narrow, forked scales – while within the opaque wing region, scale morphologies resembled ‘typical’ butterfly pigmented scales – flat and ovoid with serrations at the tips (Fig. 1K,L). The mean (±s.d.) density of scales in the adult wing were significantly lower within the clear region, with 98.2±18.1 scales per mm^2^ in males and 102.3±17.2 in females, compared with the opaque region with 374.3±22.2 scales per mm^2^ in males and 358.1+19.6 in females (*t*=−30.9, d.f.=4, *P*<0.0001 for male sample comparison, *t*=−21.9, d.f.=4, *P*<0.0001 for female sample comparison; Fig. 1N). In the clear region, forked scales were significantly smaller in size (498±39 μm^2^) compared with the bristle-like scales (831±183 μm^2^), while in the opaque region, scales were the largest (3467±382 μm^2^) (Fig. 1O). Finally, the amount of exposed wing membrane was significantly different between wing regions, with an average of 81.6±2.7 and 82.2±4.3% of exposed membrane in the clear wing regions of males and females, respectively, compared with 2.6±1.1 and 1.4±0.7% membrane exposed in opaque regions of males and females, respectively (*t*=78.9423, d.f.=4, *P*<0.0001 for male sample comparison, *t*=48.3854, d.f.=4, *P*<0.0001 for female sample comparison, Fig. 1P).

### Morphogenesis and cytoskeletal organization of developing scale cells

**Fig. 2.**
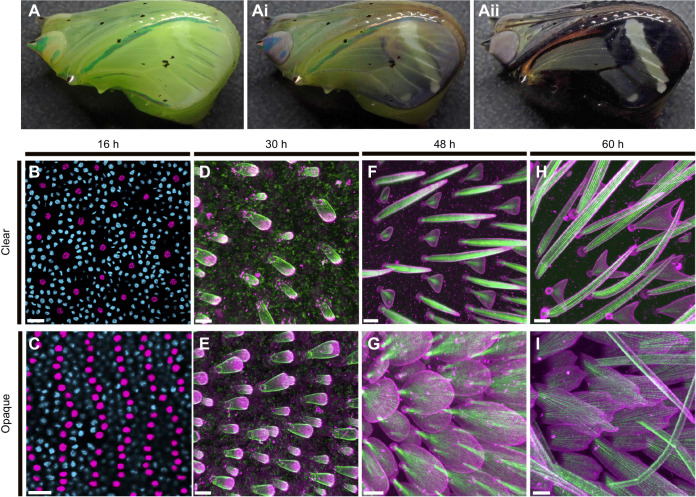
**Pupal wing development and cytoskeletal organization of scales in clear and opaque regions.** (A) Representative image of a *G.* *oto* pupa ∼5 days after pupal formation (APF), (A′,A″) developing up to the melanic stage ∼7 days APF, just prior to eclosion. (B,C) Early wing development 16 h APF stained with DAPI (nuclei) in (B) a clear wing region and (C) an opaque wing region. The clear region contains a reduced number of sensory organ precursor (SOP) cells (the precursor cells to the scale and socket cells) relative to the opaque region. Scale bars, 20 μm. SOP cells are false-colored magenta for better viewing. (D–I) Fluorescently labeled scale cell membrane (wheat germ agglutinin; WGA, magenta) and F-actin (phalloidin, green), comparing clear wing regions (D,F,H) to opaque wing regions (E,G,I). (D,E) At 30 h AFP, WGA and phalloidin staining reveal early scale buds extending from the wing epithelium and loosely organized parallel actin filaments. (F,G) At 48 h APF, scales have grown and changed in morphology. Short actin filaments have reorganized and formed smaller numbers of thick, regularly spaced parallel bundles under the cell membrane surface. (F) In the clear wing region, scale cells alternate between triangular shapes and bristles. (H,I) At 60 h APF, developing scales have become more elongated. (H) The triangular-shaped scales in the clear wing region have proceeded to generate two new branches, which fork and elongate bidirectionally. (I) In the opaque region, scales are longer and have developed serrations at the tips. Scale bars, (D–I) 10 μm.

To investigate developmental processes of wing and scale development, we performed dissections of *G. oto* pupae at different time points (Fig. 2). As in other species of Lepidoptera, the early pupal wing consisted of a thin bilayer of uniform epithelial tissue and by 16 h APF, numerous epidermal cells had differentiated to produce parallel rows of sensory organ precursor (SOP) cells (the precursors to the scale and socket cells) (Fig. 2B,C). At this early stage of wing development, we observed that the clear wing region harbored a lower density of SOP cells relative to the opaque wing region (Fig. 2B,C). In a 400 μm^2^ area, the density of SOP cells in the clear region was 65.2±7.0, compared with the density of SOP cells in the opaque region of 169.2±15.7 (*t*=−10.4629 d.f.=4, *P*=0.0003, *N*=3 pupae). We can therefore infer that early into wing development, SOP cell patterning is differentially regulated between clear and opaque regions, which impacts the adult wing scale density and the amount of wing membrane surface exposed in different parts of the wing.

Next, we investigated cellular and cytoskeletal organization during scale growth in clear and opaque wing regions (Fig. 2D–I). We found that general aspects of scale development in *G. oto* follow those previously reported in several butterfly and moth species by Dinwiddie et al. (2014), with some notable distinctions for modified scale growth in the clear wing regions of *G. oto*. By 30 h APF, the SOP cells have divided to produce the scale and socket cells (Fig. 2D,E). The scale cell body lies internally within the wing, while the socket cell associated with each scale cell lies in a more superficial position. Phalloidin staining showed the appearance of small cylindrical scale outgrowths containing F-actin filaments, and WGA staining showed outlines of the membrane as the scale outgrowths begin to project and elongate beyond the wing surface. At this stage, budding scales in the clear wing region appeared morphologically similar to the unspecialized opaque scales: roughly elongated balloon-shaped with numerous small actin rods fanning out from the pedicel to the apical tip of the scale (Fig. 2D,E). By 48 h APF, scale cell extensions have grown and elongated (Fig. 2F,G). The actin filaments have reorganized into smaller numbers of thick, regularly spaced bundles along the proximal–distal axis of the scale just under the surface of the cell membrane. Fluorescent staining revealed larger bundles of F-actin in the adwing (facing the wing membrane) side of the scales relative to the abwing side (Movie 1). At this stage, scales in different regions of the wing had taken on dramatically different morphologies. Scales in the clear region had elongated in a vertical orientation and obtained two types of alternating morphologies: short and triangular, or long and bristle-like outgrowths (Fig. 2F). In the opaque region, scales had taken on a round and flattened morphology, with ground scales shorter than the cover scales (Fig. 2G). By 60 h APF, scale projections were even more elongated (Fig. 2H,I). The triangular scales in the clear wing region had proceeded to generate two new branches, which forked and elongated at the tips bidirectionally, while bristle-like scales had elongated and curved (Fig. 2H). In the opaque region, scales were longer, wider and flatter, and had developed serrations at the tips (Fig. 2I).

### Ultrastructure analysis of developing bristle, forked and opaque scales

**Fig. 3.**
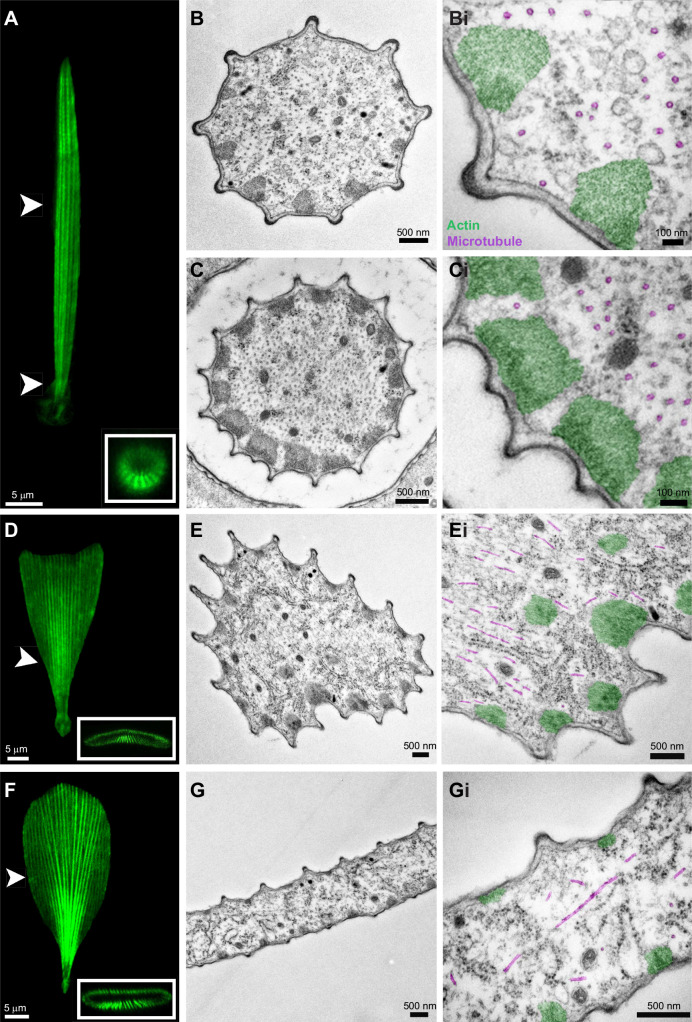
**Confocal and transmission electron microscopy (TEM) transverse sections of developing bristle (top), forked (middle) and flat (bottom) scales 48 h APF in *G. oto*.** (A) Confocal projection of a bristle-like scale morphology (phalloidin) in a clear wing region. White arrowheads show representative regions of transverse TEM sections, one near a distal region of the bristle-like scale, and one near the base of the bristle-like scale, which correspond to B and C, respectively. Scale bar, 5 μm. (B,C) TEM of a bristle-like scale in a distal region (B,B′) and a basal region near the socket cell (C,C′). Note the peripheral actin bundles (false-colored green) and internal microtubule rings (false-colored magenta). Scale bars, (B,C) 500 nm, (B′,C′) 100 nm. (D) Confocal projection of a developing forked scale (phalloidin) in a clear wing region. White arrowhead shows a representative region of transverse TEM sections. Scale bar, 5 μm. (E,E′) TEM of a forked scale reveals peripheral bundles of actin (false-colored green), with thicker actin bundles on the ventral side of the scale and internal microtubules (false-colored magenta). Two internal bundles of actin filaments can be observed in the cytoplasm (E′). Scale bars, 500 nm. (F) Confocal projections of developing flat, round scale (phalloidin) in an opaque wing region. White arrowhead shows a representative region of transverse TEM sections. Scale bar, 5 μm. (G,G′) TEM reveals asymmetry in the actin bundles (false-colored green), which are larger on the bottom side of the scale relative to the upper surface. Microtubules (false-colored magenta) are found in various orientations. Scale bars, 500 nm. The insets in A, D and F indicate confocal projections of the scales stained with phalloidin rotated horizontally.

To reveal ultrastructural detail of developing wing scale morphology, we performed TEM on pupal wing tissue of *G. oto* at 48 h APF (Fig. 3). In transverse sections, we could resolve distinct scale morphologies (bristle, forked and opaque) and their associated cytoskeletal elements.

Bristle-like scales in the clear wing regions were circular in cross-sections (Fig. 3A–C). We could also distinguish between distal and basal regions of bristle-like scales, the latter of which had the presence of a surrounding socket cell in the cross-section (Fig. 3B,C). TEM revealed that these bristle-like scales were ringed by peripheral bundles of actin filaments, which lay spaced just under the cell membrane (Fig. 3B,C′). In distal regions of the bristle-like scale, actin bundles were larger on the adwing side relative to the abwing (Fig. 3B), while near the base of the bristle-like scale (indicated by the presence of a surrounding socket cell), actin bundles were more evenly distributed around the periphery (Fig. 3C).

We also observed large populations of microtubules distributed throughout developing scales, which were internal relative to the actin bundles. Interestingly, we observed distinct patterns of microtubule distribution within different developing scale morphologies. The cross-section of bristle-like scales revealed large populations of internal microtubules, which we identified owing to their characteristic ring shape and diameter of ∼25 nm (Fig. 3B′,C′). The circular ring shape of microtubules in cross-sections of both the basal and distal parts of the bristle-like scale suggested that microtubules are all longitudinally oriented, running in the same direction as the actin filaments, parallel to growth. We also observed that populations of microtubules were localized primarily away from the surface of the scale in its interior, and microtubules were fewer distally than basally (Fig. 3B′,C′).

In our TEM cross-sections, we also observed scale types that appeared more triangular in shape, suggesting that they correspond to developing forked scales within the clear wing region (Fig. 3D,E). These scales were ringed by peripheral bundles of actin filaments, with larger actin bundles on the adwing side of the scale. Interestingly, we observed two internal bundles of actin filaments that were not observed in bristle-like scale morphologies, although we note that these could also be internal actin bundles previously referred in other butterfly species as ‘rods’, which only extend approximately two-thirds of the way along the proximal–distal axis and are only on the lower surface of the scale (Fig. 3E′) (Dinwiddie et al., 2014). We also note that there was variability in microtubule orientation, rather than the ubiquitous longitudinal orientations observed in bristle-like scales.

Finally, developing opaque scales were easily identified in cross-sections owing to their large size and flattened morphology (Fig. 3F,G). We observed peripheral bundles of actin filaments that were widely spaced and smaller in size in distal parts of the scale (Fig. 3G,G′). We observed a clear asymmetry in actin bundle size, which were larger on the adwing side of the scale relative to the abwing surface. In opaque wing regions, TEM micrographs revealed what appeared to be concentrated parallel-running populations of microtubules near the narrow base of the scales, and then a more mesh-like network of microtubules in more distal flattened regions, indicating that microtubules have varying orientations within different regions of the scale (Fig. 3G,G′, Fig. S1). In contrast to the bristle-like scales, large, flattened opaque scales appeared to contain populations of microtubules that were more widely distributed and less dense. In all scale types, we observed the presence of hexagonally packed F-actin filaments and numerous internal organelles and vesicles, including mitochondria, electron-dense vesicles and free ribosomes (Fig. 3, Fig. S1).

### Ontogeny of wing membrane nanostructures

**Fig. 4.**
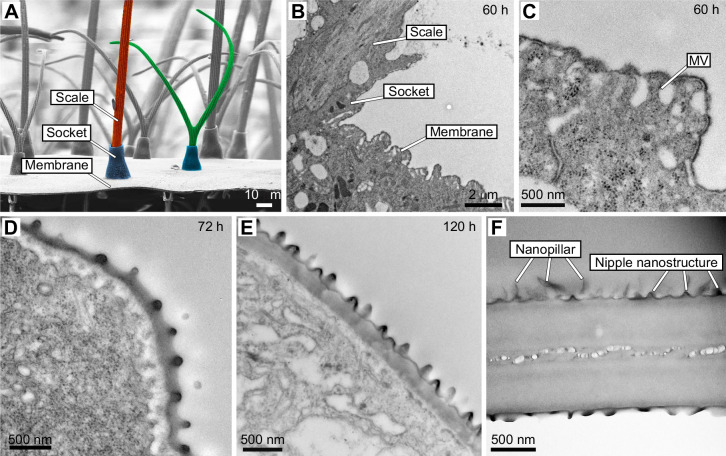
**Ontogeny of wing membrane surface nanostructures.** (A) SEM cross-section (side view) of an adult *G.* *oto* clear wing region. Scale bar, 10 μm. Bristle-like scale false colored in red, forked scale false colored in green, sockets false colored in blue. (B) TEM transverse section of epithelial tissue 60 h APF, showing lateral scale growth and wing membrane cells. Scale bar, 2 μm. (C) Higher magnification of developing wing epithelial cells at 60 h APF show microvilli (MV) projections, which appear as slender linear extensions from the inner margins of the developing cells that insert into a thin layer of electron-dense material. Lamina evaginations appear in the section as domes. (D,E) TEM of epithelial tissue (D) 72 h APF and (E) 120 h APF shows wing surface nanostructures protruding from the surface, with tips of microvilli still attached to the inner surface of the wing membrane. (F) TEM of the adult wing membrane. The surface contains dome-shaped nipple nanostructures and an upper layer of nanopillars. Scale bars, (C–E) 500 nm.

The clear wing regions of *G. oto* contain nanopillars that cover the surface of the membrane (Fig. 1I). These nanopillars were previously characterized based on SEM in adult wings, which feature an irregular height distribution and help to generate omnidirectional anti-reflective properties (Siddique et al., 2015). To gain insight into the development of these nanostructures, we examined the surface of the wing membrane epithelial cells with TEM (Fig. 4B–F). At 60 h APF, a perpendicular section through the wing epithelia showed a continuous epithelial lamina (Fig. 4B,C). We observed that the epithelial cells contained microvilli, which appeared as slender linear extensions from the inner margins of the developing cells that insert into electron-dense material (Fig. 4B,C). The surface layer of the epithelia appeared as an extracellular lamellar system, and lamina evaginations appeared in the section as domes distal to the microvillar extensions (Fig. 4C). By 72 h APF, we observed a thin outer layer of the epicuticle that rose above the epidermal cells, and by 120 h APF, we found that this layer above the microvilli contained what appear to be dome-shaped protrusions and thickened cuticle, possibly secreted from regularly spaced microvilli (Fig. 4D,E). Finally, in our TEM cross-section of a fully developed adult wing of *G. oto*, we observed that the membrane surface harbors dome-shaped nanoprotrusions with morphologies similar to those of insect corneal surface nipple arrays (Yoshida et al., 1997; Bernhard, 1962), which we refer to throughout the text now as ‘nipple nanostructures’, and an upper layer containing pillar-like protrusions, which we refer to as ‘nanopillars’, that featured a more irregular height distribution (Fig. 4F). These results show early subcellular processes of developing nanopillars within the clear wing region, which arise distal to microvillar extension in epithelial cells.

### Topographical organization and biochemical composition of wing surface nanostructures

**Fig. 5.**
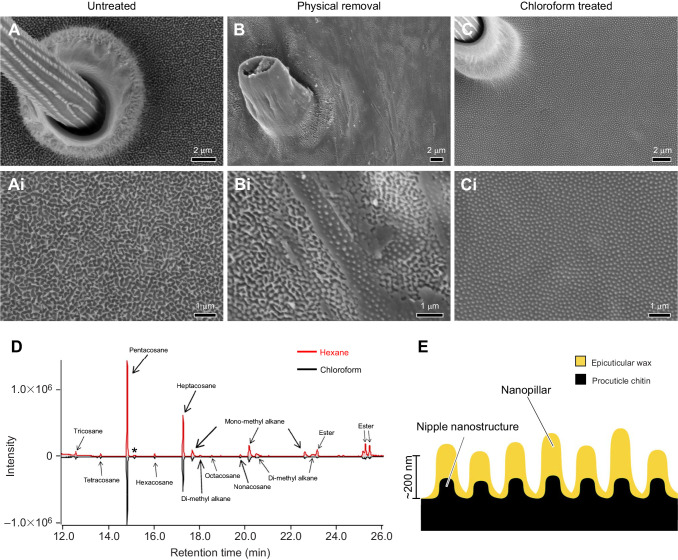
**Topographical organization and biochemical composition of wing surface nanostructures.** SEM of the transparent wing membrane surface of *G.* *oto* under (A,A′) the untreated condition, highlighting the presence of irregularly arranged nanopillar structures covering the surface, (B,B′) the physical treated condition, revealing partial removal of surface nanopillars, and a lower layer of more regularly arranged nipple-like nanostructures and (C) the chloroform-treated condition, revealing complete removal of the upper layer of nanopillars, and remaining lower layer of nipple-like nanostructures. Scale bars, (A–C) 2 μm, (A′–C′) 1 μm. (D) Chromatogram of hexane-treated (top; red line) and chloroform-treated (bottom; black line) clearwing extracts. *x*-axis shows the retention time in minutes and *y*-axis shows the abundance of total ion current. (E) Schematic of proposed wing surface membrane nanostructures in *G.* *oto*, composed of chitin-based procuticle and wax-based epicuticle.

Based on our electron microscopy results of membrane nanostructures, we investigated the topographical organization and biochemical composition of the adult wing surface. To do so, we treated individual, disarticulated adult *G. oto* wings in two ways: (1) by physically removing wing surface nanostructures by gently pressing and rubbing a wing in between paper and Styrofoam (Yoshida et al., 1997) and (2) by testing the wing surface structures for solubility in organic solvents, including hexane and chloroform to extract lipids (Futahashi et al., 2019). We then performed SEM to compare wing surface topography of untreated and treated wing samples (Fig. 5A–C′). SEM confirmed that the first treatment partially or completely removed nanostructures across the wing membrane surface (Fig. 5B). In a region of partial removal, we could identify smaller, dome-shaped nipple nanostructures underneath the top layer of nanopillars (Fig. 5B′). SEM of the chemically treated wing surface revealed that the upper layer of irregularly sized nanopillars was completely removed, revealing a layer of regularly arranged dome-shaped nipple nanostructures that did not dissolve through chloroform or hexane exposure (Fig. 5C,C′). Therefore, we hypothesized that the upper layer of irregularly sized nanopillars consisted of a secreted wax-based material, which sits above smaller chitin-based nipple nanostructures.

To test this hypothesis, we extracted the surface layer of *G. oto* clear wing regions with either hexane or chloroform and analyzed the chemical composition by gas chromatography–mass spectrometry (GC-MS). We found that the chemical profile generated by both hexane and chloroform extracts yielded similar results (Fig. 5D). In all extracts, we identified two straight-chain alkanes that made up approximately two-thirds of the compounds detected: 41.64±5.75% pentacosane (C_25_H_52_) and 23.32±5.35% heptacosane (C_27_H_56_) (Table S1). The remaining compounds were primarily composed of slightly larger methyl-branched alkanes (monomethyl and dimethyl C27, C29 and C31) and esters. Therefore, our results suggest that in *G. oto*, there are two components to wing surface ultrastructure: procuticle-based nipple nanostructures, and an upper epicuticular layer of irregularly sized nanopillars, composed mainly of straight-chain alkanes (Fig. 5D,E).

### Anti-reflective properties of wax-based nanopillars

**Fig. 6.**
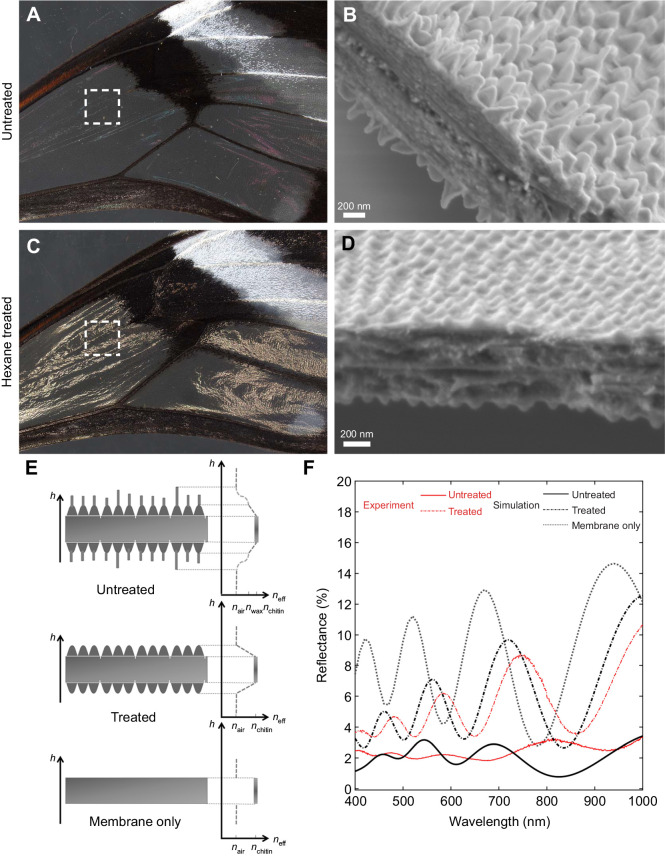
**Structural elements, reflectance spectra and optical modeling of anti-reflective nanostructures.** Optical images and cross-section SEM of *G.* *oto* (A,B) untreated wings, illustrating low reflectance and the presence of nanopillars on the wing membrane surface, and (C,D) hexane-treated wings, illustrating increased reflectance and the loss of nanopillars on the wing membrane, but presence of nipple-like nanostructures on the surface. The dashed squares in A and C indicate approximate regions of the wing used for SEM and spectral reflectance measurements. Scale bars, (B,D) 200 nm. (E) Optical modeling of effective refractive index conditions for (top) untreated wings, with nanopillars of variable height together with cuticle-based nipple nanostructures on the wing membrane, (middle) treated wings, with cuticle-based nipple nanostructures on wing membrane, and (bottom) wing membrane without any nanostructure. *y*-axis represents height *h* and *x*-axis represents effective refractive index condition of air (*n*_air_), chitin (*n*_chitin_) and wax (*n*_wax_). (F) Representative reflectance spectra of experimental (red) and simulation data (black) for untreated wings with nanopillars on the membrane surface (solid line), hexane-treated wings with the wax-based layer of nanopillars removed (dashed line) and membrane only (dotted line).

To address whether the wax-based nanopillars play a role in wing reflection, we measured the reflectance spectra of untreated and hexane-treated wings (Fig. 6). Additionally, we measured nanostructure geometries and membrane thickness from wing SEM cross-sections and determined the average distance between two nanostructures as *d*=174 nm, conical-shaped cuticular nipple nanostructure height as *h*_p_=77 nm, wax-based irregular nanopillar radius as *r*_np_=53 nm, mean height as *h*_np_=224 nm and variance as σ_np_=49.3 nm, and membrane thickness as *h*_m_=746 nm and variance as σ_m_=43 nm (Fig. 6B,D, Fig. S2). On the basis of SEM micrographs for treated and untreated samples, we modeled three wing architectures, consisting of: (1) nanopillars with variable height together with cuticle-based nipple nanostructures on the wing membrane, (2) cuticle-based nipple nanostructures on the wing membrane and (3) the wing membrane without any nanostructures, to simulate the optical properties for different conditions (Fig. 6E). The simulated reflectance data of the untreated and treated conditions in Fig. 6F closely resembled the experimental ones. In untreated wings of *G. oto*, we found that transparent regions have a low total diffuse reflection of approximately 2%, which is in line with previous reflectance measurements of this species (Siddique et al., 2015) (Fig. 6F). By contrast, the hexane-treated wings without the upper layer of wax nanopillars had approximately 2.5 times greater reflectance relative to the untreated wings, and generated an iridescent thin film spectra, even though they harbored dome-shaped nipple nanostructures (Fig. 6D,F).

For simulated data, the overall reflectance ratio of the hexane-treated wing to the untreated wing was approximately three, similar to experimental reflectance data (Fig. 6F; see dataset available from Dryad at https://doi.org/10.6078/D1TD7H). Importantly, the simulated results for the untreated wing with wax-based irregular nanopillars make reflectance more uniform across wavelengths, which reduces the iridescent effect of the wing membrane. Finally, we simulated a thin film membrane without any nanostructures, which showed reflectance (averaged from all wavelengths) of the membrane itself to be 8.81±3.46%, whereas the treated and untreated wing reflections were 5.78±2.82% and 1.93±0.77%, respectively (Fig. 6F). While treated wings harboring dome-shaped nipple nanostructures reduced the overall reflectance relative to the membrane only, their effect was not strong enough to reduce reflectance spectra oscillation. The wax-based irregular nanopillars on top introduced a more gradual transition between refractive indices to lessen the oscillation by approximately five-fold, in addition to reducing overall reflection (Fig. 6F). Additionally, we simulated the three wing architecture models considering different mean membrane thicknesses and variance in membrane thickness (Fig. S3). We found that variance in wing membrane thickness reduced reflectance spectra oscillations, rather than mean membrane thickness alone, and more peaks appear in the visible spectrum with increasing thickness of the membrane. (Fig. S3; Dryad dataset https://doi.org/10.6078/D1TD7H). Overall, these results demonstrate that the non-constant architecture of the wing membrane and wax-based irregular nanopillars on the wing surface of *G. oto* function to dramatically enhance anti-reflective properties.

### Solubility of wing surface nanostructures in clearwing Lepidoptera

**Fig. 7.**
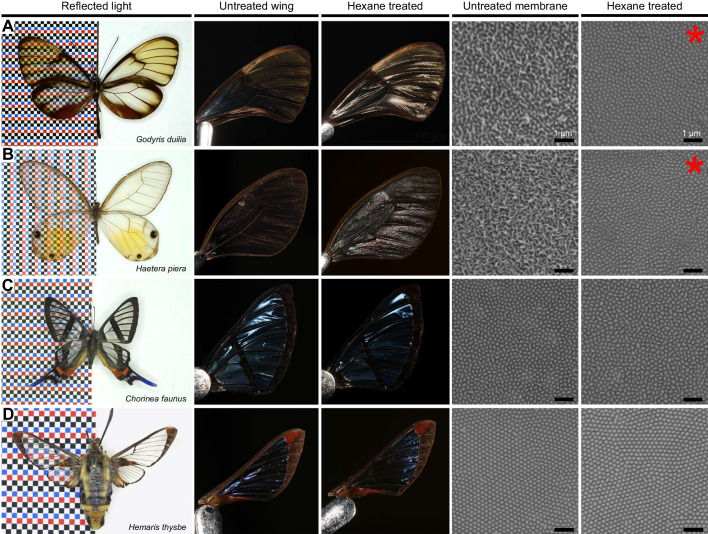
**Solubility of wing surface nanostructures in additional species of clearwing Lepidoptera.** Untreated wings, hexane-treated wings, SEM of untreated membrane and SEM of hexane-treated membrane for (A) *Godyris duilia* (Nymphalidae: Ithomiini), (B) *Haetera piera* (Nymphalidae: Haeterini), (C) *Chorinea faunus* (Riodinidae: Riodinini) and (D) *Hemaris thysbe* (Sphingidae: Dilophonotini). For both (A) *G. duilia* and (B) *H. piera*, the membrane surface contains irregularly arranged nanopillar structures. After hexane treatments, the wings become more reflective and the upper layer of irregularly arranged nanopillars is removed, while nipple-like structures remain (indicated by red asterisks). For both (C) *C. faunus* and (D) *H. thysbe*, the reflectivity of the wings and the regularly arranged nipple array-like nanostructures on the membrane surface appear unaffected after hexane treatment. Scale bars, 1 μm.

We investigated additional species of clearwing Lepidoptera by assessing the solubility of wing surface nanostructures with hexane treatments, including (A) an additional glasswing butterfly, *Godyris duilia* (Nymphalidae: Ithomiini), (B) the amber phantom butterfly, *Haetera piera* (Nymphalidae: Haeterini), (C) the longtail glasswing, *Chorinea faunus* (Riodinidae: Riodinini), and (D) the clearwing hawkmoth, *Hemaris thysbe* (Sphingidae: Dilophonotini) (Fig. 7). For both *G. duilia* and *H. piera*, we found that the clear wing membrane surface is covered in irregularly arranged nanopillar structures (Fig. 7A,B). After hexane treatments, the wings became more reflective, the upper layer of irregularly arranged nanopillars was removed, while nipple-like structures remained, supporting that nanopillars are likely wax-based, similar to *G.* *oto.* Conversely, for both *C. faunus* and *H. thysbe*, the reflectivity of the wings and the regularly arranged nipple array-like nanostructures on the membrane surface appeared unaffected after hexane treatment, suggesting that the structures are chitin-based (Fig. 7C,D). These results indicate that wing surface nanostructures can be either chitin-based, which morphologically resemble the nipple array type of nanostructure, or wax-based, which morphologically resemble irregularly arranged nanopillars, and both types appear to have arisen in phylogenetically distant lineages of Lepidoptera.

## DISCUSSION

Butterflies and moths have evolved sub-wavelength anti-reflective structural innovations on their wings that enable them to be transparent. Here, we report the details of pupal wing development and scale cytoskeletal organization in the glasswing butterfly, *G.* *oto*, as well as insights into the ontogeny and biochemical basis of wing surface nanostructures that reduce reflection in clearwing Lepidoptera.

The arrangement of unicellular projections in insect integument, such as bristles and scales, has been a model for research on cellular pattern formation (Ghiradella and Butler, 2009). Shortly after pupation, SOP cells develop from a monolayer of epithelial cells into orderly arrangements, then differentiate into scale and socket cells. In the present study, we found that early SOP cell patterning affects the final adult scale density in *G. oto*, and this feature of spacing scale cells farther apart, and therefore reducing the overall density of scales, is an initial step to generate clear wings. During early pupal development, the receptor molecule Notch is expressed in a grid-like pattern in the wing epithelium (Reed, 2004). This may contribute to the parallel rows of uniformly spaced SOP cells, which express a homolog of the *achaete-scute* proneural transcription factors that likely function in scale precursor cell differentiation (Galant et al., 1998). Notch-mediated lateral inhibition could establish a dense population of ordered SOP cells in the developing wing, resulting in a characteristic ratio of scale-building and epithelial cells (Escudero et al., 2003; Couturier et al., 2019). Future studies should investigate whether modifications in Notch signaling play a role in scale cell patterning in clearwing butterflies and moths, many of which contain reduced densities of scale cells (Gomez et al., 2020 preprint; Pinna et al., 2020 preprint).

The range of morphological diversity among scales and bristles within Lepidoptera likely results developmentally from components or modifiers of the cytoskeletal structures and cell membrane. One study surveyed a wide range of developing butterfly and moth scales and identified that F-actin is required for several aspects of scale development, including scale cell elongation and proper orientation (Dinwiddie et al., 2014). In the developing bristle-like scales in *G. oto*, we find relatively symmetrical actin bundles distributed throughout the periphery and a large population of longitudinally running interior microtubules. This is similar to what has been described for developing bristles in *Drosophila melanogaster* pupae, which contain peripheral bundles of cross-linked actin filaments and a large population of microtubules that run longitudinally along the bristle (Tilney et al., 2000). It was recently shown that actin bundles play different roles in shaping scales and bristles in the mosquito *Aedes aegypti*, in which developing bristles contained symmetrically organized actin bundles, while actin bundle distribution in scales became more asymmetrically organized (Djokic et al., 2020). Given that actin dynamics play a variety of roles in regulating the development of bristles and scales (Dinwiddie et al., 2014; Day et al., 2019; Tilney et al., 2000; Djokic et al., 2020), we hypothesize that modifications in F-actin organization of scales in the transparent wing of *G. oto* are responsible in part for their narrow bristle-like and forked morphologies.

In an analysis of moth scale development, major shape changes were found to be correlated with changes to the orientation of the cytoplasmic microtubules (Overton, 1966). In the present study, we identified large populations of microtubules organized throughout developing scales and found that microtubules exhibit different distributions and orientations relative to distinct scale morphologies, namely between bristle, forked and flat, round scales. In *D. melanogaster*, microtubules may play a role in bristle development by adding bulk to the bristle cytoplasm, contributing to proper axial growth, and aiding organelle and protein distribution (Bitan et al., 2010, 2012). It would be interesting for future studies to functionally characterize the role microtubules play in the development of lepidopteran scales. Our findings lend further support to the observations that general patterns of scale development, including patterns of F-actin localization and microtubule distribution, seem to be well conserved in Lepidoptera, and that modifications of scale morphology to achieve clearwing phenotypes, such as narrow bristle-like and forked scales, likely involve alteration of cytoskeletal organization during scale growth.

Chitinous wing membrane has a higher refractive index than air, which generates glare under natural light conditions. Some clearwing species have evolved sub-wavelength anti-reflective nanostructures, which reduces glare and likely aids in crypsis (Yoshida et al., 1997; Siddique et al., 2015). In this study, we identified the early developmental processes of nanostructures that arise in the wing epithelium. We also note interesting parallels of our observations to previous descriptions of developing nanostructures on the surface of insect cornea. Early data on pupal development of corneal nanostructures were produced by detailed electron microscopy studies, showing that corneal nipples emerge during lens formation (Gemne, 1971; Fröhlich, 2001). In these observations, development of initial laminar patches formed on top of underlying microvilli. Subsequently, nanostructures (termed nipple structure array) formed on the surface, with the tips of microvilli still attached to the inner surface. Gemne (1971) proposed that the corneal nanostructures originate from secretion by the regularly spaced microvilli of the cone lens cells, although there is still debate about the exact nature of how microvilli pre-pattern nanostructure arrays (Kryuchkov et al., 2017). Our TEM results provide insight into the early developmental processes of anti-reflective nanostructure formation in the wings of *G. oto*, highlighting certain similarities to nipple array development in insect cornea. It would be interesting for future work to explore whether features of nanostructure formation arose independently in insect cuticle as a mechanism to reduce surface reflection.

In contrast to previously described highly ordered nipple arrays found on insect eyes and some clearwing lepidopteran wings (Stavenga et al., 2006; Kryuchkov et al., 2017), the irregularly sized anti-reflective nanopillars in the clear regions of *G. oto* wings appear to consist of an upper layer of wax-based epicuticle sitting above procuticle-based nipple nanostructures. Insect cuticle is an extracellular matrix formed by the epidermis and is composed of three layers: the outermost envelope, the middle epicuticle and the inner procuticle (Moussian, 2010). The envelope and the epicuticle are composed mainly of lipids and proteins, while the procuticle contains the polysaccharide chitin. Many terrestrial arthropods deposit a layer of wax lipids on the surface of their cuticle, which reduces evaporative water loss (Gibbs, 1998). In some species of dragonfly, epicuticular wax-based nanostructures have also been demonstrated to play a role in generating optical properties, such as an ultraviolet reflection (Futahashi et al., 2019). In mature males of these dragonflies, a dense wax secretion composed of long-chain methyl ketones, in particular 2-pentacosanone, was found to contribute to the UV reflection properties (Futahashi et al., 2019). The chemical composition of nanopillars on the wing surface of cicadas, which contribute to hydrophobicity and antimicrobial properties, was found to consist of epicuticular components such as fatty acids and hydrocarbons ranging from C_17_ to C_44_ (Román-Kustas et al., 2020). Another study exploring the molecular organization of dragonfly wing epicuticle found that the major components identified were fatty acids and *n*-alkanes with even-numbered carbon chains ranging from C_14_ to C_30_ (Ivanova et al., 2013). Here, we identified that the epicuticular layer of irregularly sized anti-reflective nanopillars in *G. oto* appears to be composed mainly of *n*-alkanes, including pentacosane (C_25_) and heptacosane (C_27_) and showed the importance of these structures in attaining better transparency. Interestingly, we found that butterflies belonging to the tribe Haeterini also contain irregularly arranged hexane-soluble nanopillars on the wing membrane surface, suggesting that wax-based anti-reflective structures have arisen multiple times independently.

Turing reaction–diffusion mechanisms have been proposed as a model for the formation of various corneal nanostructure morphologies (such as spacing, height, and spatial organization) during insect eye development (reviewed in Kryuchkov et al., 2017). Although the degree of height irregularity of nanopillars is important for achieving omnidirectional anti-reflection in *G. oto*, we do not yet understand how such variability in height is generated. Perhaps the pressure of the wax secretion varies across the area of microvillar extensions, similar to how nozzle area plays a role in the propulsion force, and tunes the height of the nanopillars in the process. In such a scenario, the degree of the height variation could be synthetically engineered depending on the two-dimensional nanopatterned mask design in the biomimetic processes, such as molding or imprinting techniques. Additionally, others have generated three-dimensional wax structures using *n*-alkanes, noting that wax-based crystals can generate different shapes, sizes and densities depending on the chain length (Gorb et al., 2014). Future work should investigate the possible role of alkanes, and the two-dimensional surface growth geometry, in generating three-dimensional anti-reflective nanostructures and potential applications for biomimetics.

Taken together, these results enable us to form a hypothesis that the origin of anti-reflective nanopillars may have involved a two-step evolutionary process. First, regions of wing membrane may have become increasingly exposed through a reversion of dense, flat, wing scales to fewer, narrow more bristle-like scales. Next, membrane surface nanostructures may have arisen and reduced surface reflection, which became an advantageous phenotype owing to enhanced crypsis and reduced predation. Interestingly, some basal ithomiines contain nanostructures on the membrane surface, despite having opaque wings (C.P., unpublished observations). Wing surface nanostructures are also known to provide antibacterial and hydrophobicity properties in insects, which may explain why they are present in some opaque species. This presents an interesting question of whether wing surface nanostructures in clearwings were already present in an opaque ancestor and were selected for anti-reflective properties, or whether they arose *de novo*. In either scenario, this potential two-step evolutionary process may have required different sets of developmental programs or gene networks that co-occurred to generate wing transparency. Future studies of scale and nanostructure development and evolutionary histories of transparent species and their opaque ancestors will help to elucidate how transparency repeatedly arose in Lepidoptera. Our exploration of *G.* *oto* wing development can serve as a model for understanding how transparent phenotypes evolved within Ithomiini, a diverse tribe of neotropical butterflies that act as mimicry models for numerous species of Lepidoptera Elias et al. (2008), as well as more distantly related butterfly and moth species.

## Supplementary Material

Supplementary information
