# Supplementary material for: Developmental, cellular and biochemical basis of transparency in clearwing butterflies
Source: J Exp Biol. 2021 May 28;224(10):jeb237917. doi: 10.1242/jeb.237917 (PMC8340268; doi:10.1242/jeb.237917)
Supplement: Supplementary information [file jexbio-224-237917-s1.pdf]

Table S1 . GC-MS relative proportions (mean ± standard deviation) of wing cuticular compounds isolated from *Greta oto*.

Peak	Compound	Retention time mean (min)	Retention index*	Hexane treatment (n=2) mean ± SD (%)	Chloroform treatment (n=2) mean ± SD (%)	Combined (n=4) mean ± SD (%)
1	Tricosane	12.57	2300	1.31 ± 0.29	1.42 ± 0.00	1.36 ± 0.18
2	Tetracosane	13.66	2400	0.90 ± 0.32	1.07 ± 0.01	0.98 ± 0.21
3	Pentacosane	14.82	2500	38.28 ± 7.27	45.00 ± 1.11	41.64 ± 5.75
4	Hexacosane	16.03	2600	1.33 ± 0.33	1.48 ± 0.00	1.41 ± 0.21
5	Heptacosane	17.28	2700	19.81 ± 5.67	26.84 ± 2.12	23.32 ± 5.35
6	13-, 11-, 9-, and 7-methylheptacosane	17.68	2732	2.13 ± 3.01	3.22 ± 2.25	2.68 ± 2.26
7	x,y-dimethylheptacosane(s)	18.03	2762	0.35 ± 0.49	0.37 ± 0.52	0.36 ± 0.41
8	Octacosane	18.50	2800	0.36 ± 0.06	0.50 ± 0.28	0.43 ± 0.18
9	Nonacosane	19.80	2900	1.01 ± 0.42	1.05 ± 0.06	1.03 ± 0.25
10	15-, 13-, 11-, 9-, and 7-methylnonacosane	20.18	2929	3.50 ± 4.96	4.44 ± 3.99	3.97 ± 3.71
11	x,y-dimethylnonacosane(s)	20.48	2052	1.07 ± 1.51	0.71 ± 1.00	0.89 ± 1.07
12	15-, 13-, 11-, 9-, and 7-methylhentriacontane	22.62	3122	1.67 ± 2.36	1.68 ± 1.42	1.67 ± 1.59
13	x,y-dimethylhentriacontane(s)	22.95	3150	0.58 ± 0.82	0.02 ± 0.03	0.30 ± 0.58
14	x,y-dimethylhentriacontane(s)	23.02	3155	0.62 ± 0.51	0.00 ± 0.00	0.31 ± 0.46
15	Ester	23.17	3168	2.77 ± 1.18	1.57 ± 0.45	2.17 ± 1.01
16	Undetermined	25.19	3339	3.92 ± 1.48	2.57 ± 0.01	3.24 ± 1.16
17	Ester	25.29	3348	5.74 ± 1.72	2.87 ± 1.32	4.30 ± 2.08
18	Ester	25.47	3364	4.66 ± 1.23	2.40 ± 1.15	3.53 ± 1.63
19	Undetermined	27.54	3549	1.16 ± 0.22	0.19 ± 0.13	0.67 ± 0.58
20	Undetermined	27.71	3564	1.50 ± 0.02	0.44 ± 0.63	0.97 ± 0.71
21	Undetermined	29.80	3762	3.04 ± 0.54	1.17 ± 1.24	2.11 ± 1.33
22	Undetermined	30.02	3782	1.24 ± 0.12	0.33 ± 0.46	0.78 ± 0.59
23	Undetermined	30.81	3860	0.61 ± 0.17	0.07 ± 0.10	0.34 ± 0.33
24	Undetermined	31.82	3960	1.77 ± 1.12	0.45 ± 0.64	1.11 ± 1.06
25	Undetermined	32.03	3981	0.69 ± 0.23	0.15 ± 0.21	0.42 ± 0.36

* Non-isothermal Kovats retention indices (Van den Dool and Kratz 1963)

Journal of Experimental Biology • Supplementary information

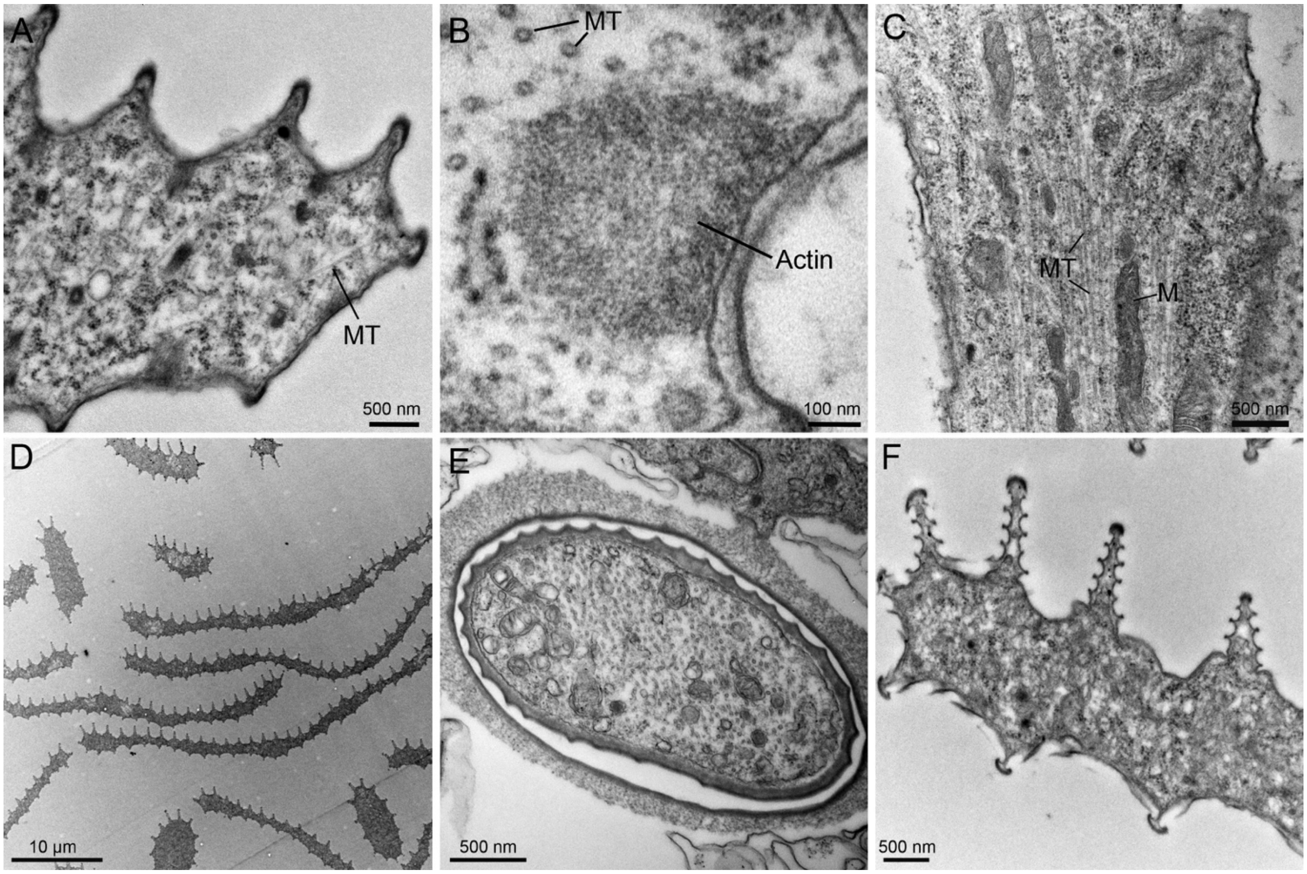

Fig. S1. TEM micrographs of scales 72 hours (top) and 120 hours (bottom) after pupal formation

(A) TEM micrograph of a developing opaque scale 72 h APF, highlighting microtubule arrangement (MT). **(B)** Thick actin bundles contain dense, hexagonally packed F-actin filaments. **(C)** Basal region of a developing scale outgrowth and socket cell. Developing scales 72 h APF contain dense populations of microtubules (MT) and numerous internal organelles, including mitochondria (M), electron dense vesicles and free single ribosomes. **(D)** Transverse section of developing scales around 120 h APF, highlighting both flat and thin, bristle-like scale morphologies. Cross section near the **(E)** base and **(F)** distal region of scales 120 h APF, showing thickened cuticle and ridge morphologies.

Journal of Experimental Biology • Supplementary information

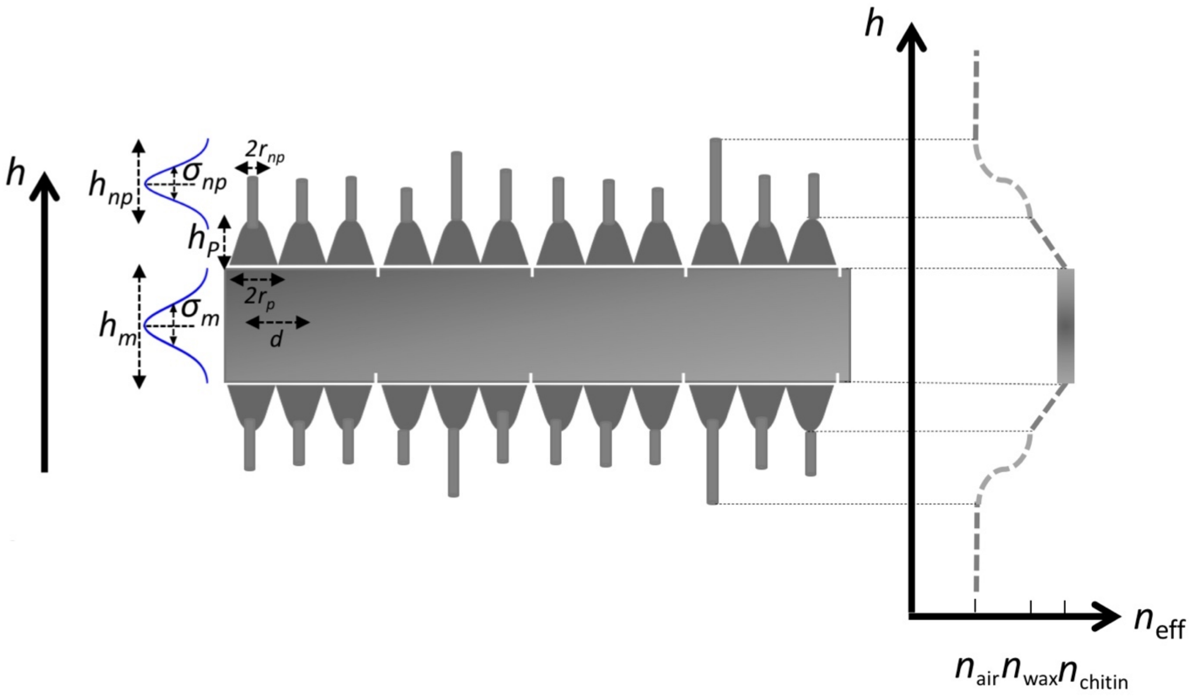

Fig. S2. Optical modeling parameters and effective refractive index condition for untreated transparent wing of *Greta oto*

Schematic representation for the optical modeling parameters of wing membrane and surface nanostructures. Average distance between two nanostructures represented as d, conical shaped cuticular nipple nanostructures height as h_p, wax-based irregular nanopillars radius as r_{np}, mean height as h_{np} and variance σ_{np}, and membrane thickness as h_m and variance σ_m. Y-axis represents height h and X-axis represents effective refractive index condition of air (n_{air}), chitin (n_{chitin}), and wax (n_{wax}).

Journal of Experimental Biology • Supplementary information

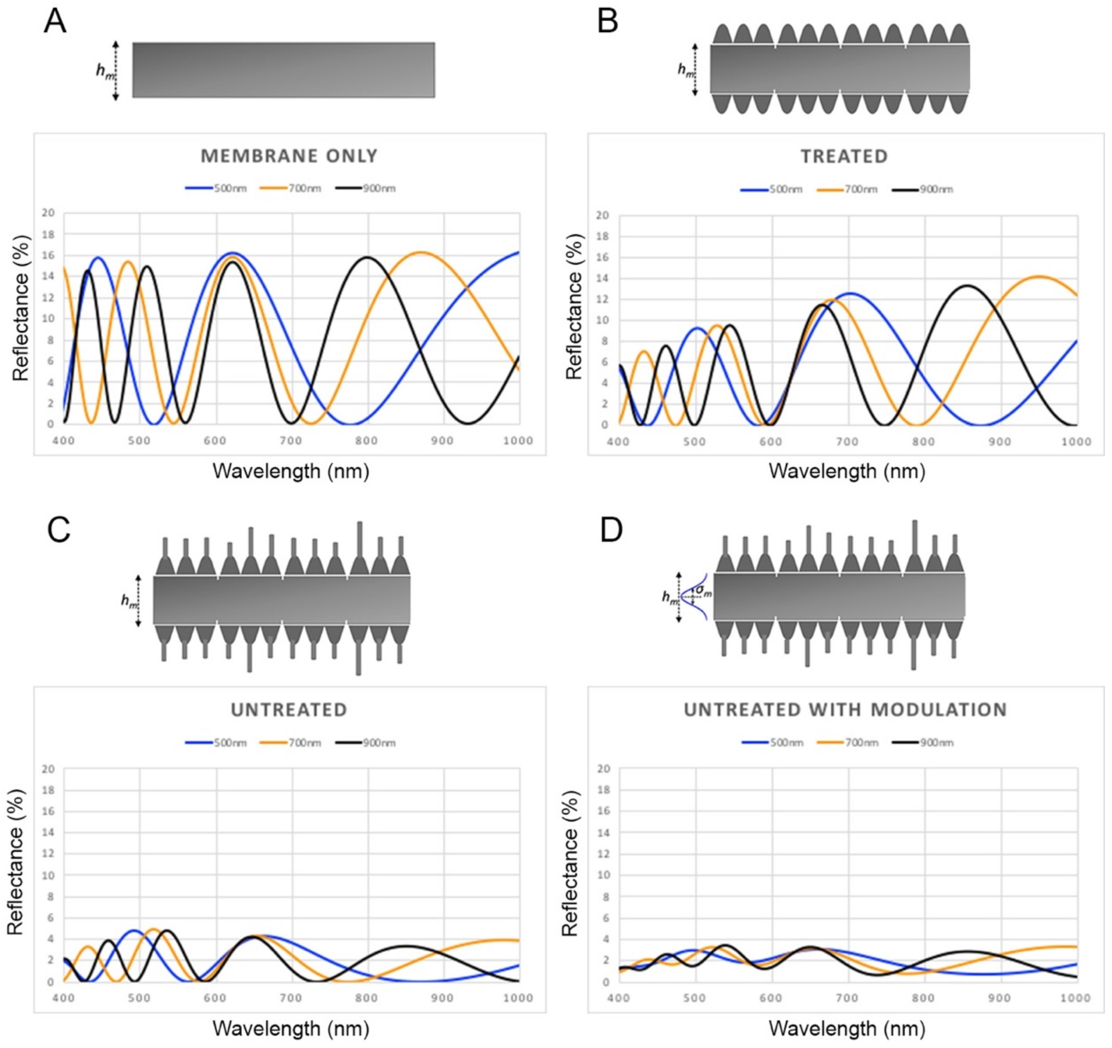

Fig. S3. Optical simulations for mean membrane thickness and modulation of thickness under different wing architecture models

Simulation reflectance spectra of **(A)** Membrane only (lacking surface nanostructures) with varying mean membrane thickness. **(B)** Treated wings (containing cuticle-based nipple nanostructures but lacking wax-based irregular nanopillars) with varying mean membrane thickness. **(C)** Untreated wings (containing wax-based irregular nanopillars and nipple nanostructures) with varying mean membrane thickness and no modulation in thickness. **(D)** Untreated wings with variable mean membrane thickness and modulation of 43 nm variance in thickness.

Journal of Experimental Biology • Supplementary information

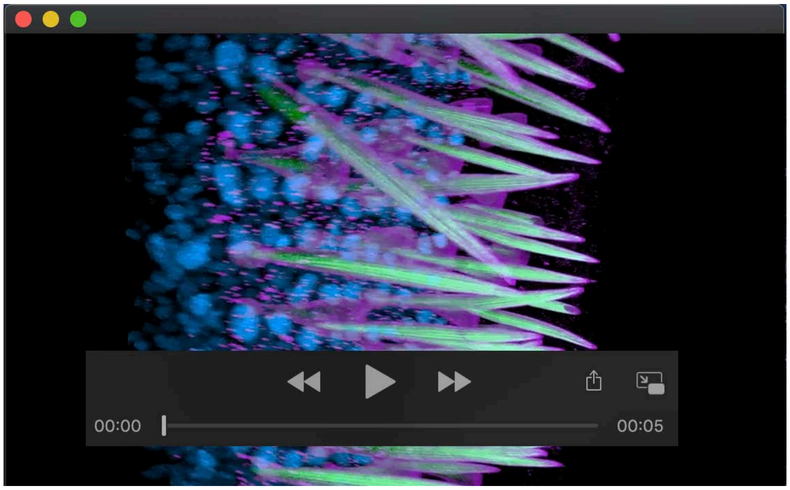

Movie 1. 3D projection of developing scales in a clear wing region 48 hours after pupal formation.

3D projection and rotation of the same scales shown in Fig. 2F, 48 hours APF in a clear wing region. WGA (magenta) stains cell membranes and phalloidin (green) stains F-actin and DAPI (blue) stains nuclei. Short actin filaments have reorganized and formed smaller numbers of thick, regularly spaced parallel bundles just under the surface of the cell membrane. Scales alternate with future forked scales appearing as triangular shapes and longer future bristle-like shapes.

Journal of Experimental Biology • Supplementary information
